# Peste des petits ruminants (PPR): A neglected tropical disease in Maghreb region of North Africa and its threat to Europe

**DOI:** 10.1371/journal.pone.0175461

**Published:** 2017-04-20

**Authors:** Ratiba Baazizi, Mana Mahapatra, Brian Donald Clarke, Khatima Ait-Oudhia, Djamel Khelef, Satya Parida

**Affiliations:** 1 National Veterinary Higher School, Algiers, Algeria; 2 The Pirbright Institute, Woking, Surrey, United Kingdom; Institut National de la Recherche Agronomique, FRANCE

## Abstract

**Background:**

Peste des petits ruminants (PPR) is a contagious disease listed by the World Organisation for Animal health (OIE) as being a specific hazard. It affects sheep, goats, and wild ungulates, and is prevalent throughout the developing world particularly Asia, the Middle East, and Africa. PPR has been targeted for eradication by 2030 by the Food and Agriculture Organization of the United Nations (FAO) and the OIE, after the successful eradication of the related disease, rinderpest in cattle. PPR was first reported in 1942 in the Ivory Coast in Western Africa and has since extended its range in Asia, the Middle East, and Africa posing an immediate threat of incursion into Europe, South East Asia and South Africa. Although robust vaccines are available, the use of these vaccines in a systematic and rational manner is not widespread, resulting in this devastating disease becoming an important neglected tropical disease in the developing world.

**Methodology:**

We isolated and characterized the PPR virus from an outbreak in Cheraga, northern Algeria, during October 2015 by analyzing the partial N-gene sequence in comparison with other viruses from the Maghreb region. As well as sequencing the full length viral genome and performing real-time RT-PCR on clinical samples. Maximum-likelihood and Bayesian temporal and phylogeographic analyses were performed to assess the persistence and spread of PPRV circulation from Eastern Africa in the Maghreb region of North Africa.

**Conclusions:**

Recent PPR outbreaks in Cheraga, in the northern part of Algiers (October 2015) and North-West Morocco (June, 2015) highlight that PPRV has spread to the northern border of North Africa and may pose a threat of introduction to Europe. Phylogeographic analysis suggests that lineage IV PPRV has spread from Eastern Africa, most likely from the Sudan 2000 outbreak, into Northern Africa resulting in the 2008 Moroccan outbreak. Maximum-likelihood and Bayesian analysis shows that these North African viruses cluster closely together suggesting the existence of continual regional circulation. Considering the same virus is circulating in Algeria, Morocco and Tunisia, implementation of a common Maghreb PPR eradication strategy would be beneficial for the region.

## Introduction

**Fig 1:**
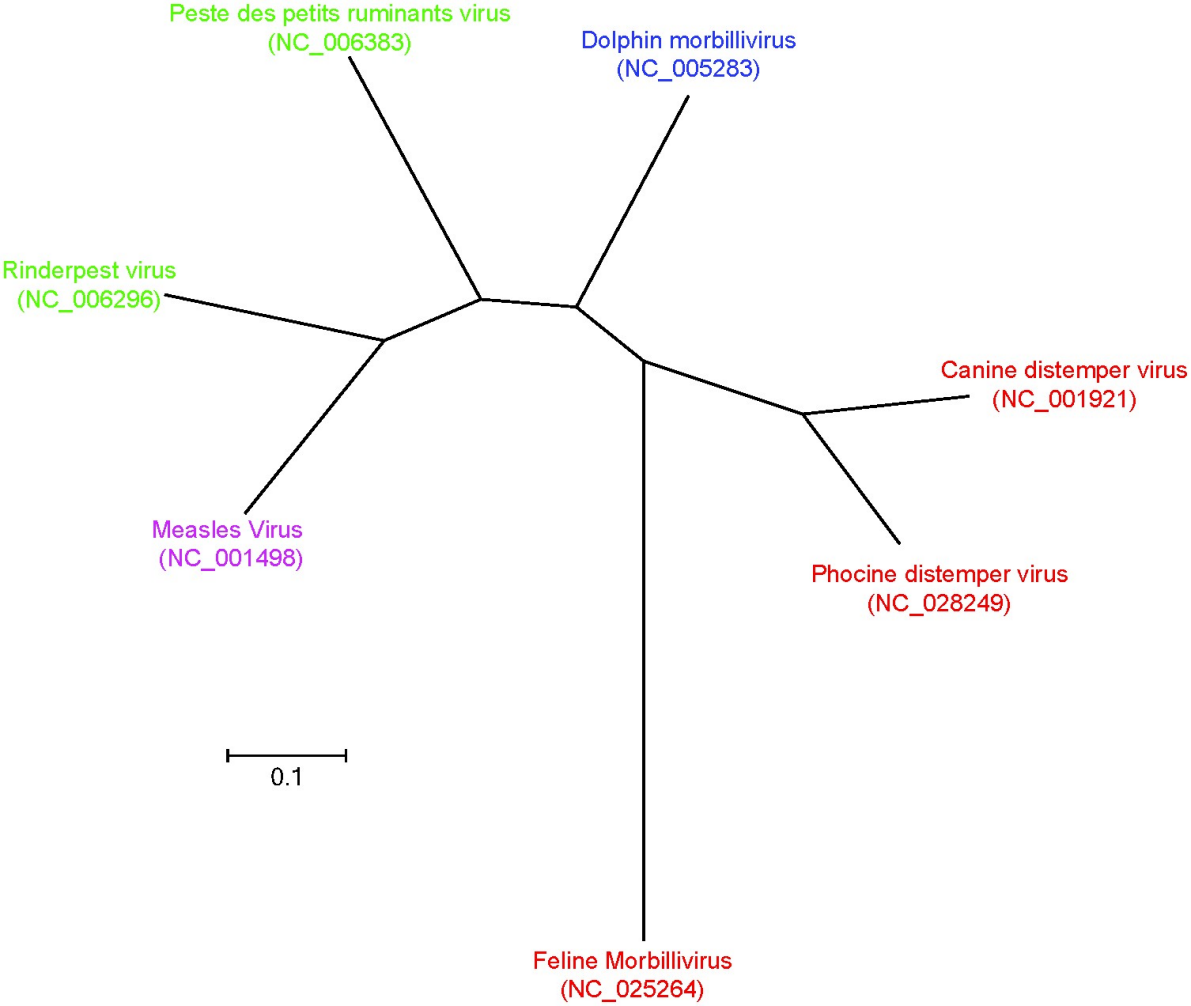
Phylogenetic tree based on full length reference sequences of members of the morbillivirus group. GenBank accession numbers are included in brackets, and primary host groups indicated by ruminants (green), cetacean (Blue), human (Pink), and carnivores (Red).

Peste des petits ruminants (PPR), is a highly contagious viral disease of small ruminants which was first reported in Cote-d’Ivoire in 1942 [1], and the disease was observed progressively further east during the eighties through East Africa, the Middle East, and Asia [2]. The causative agent of PPR; the *morbillivirus* peste-des-petits ruminants virus (PPRV), is a small single-stranded negative-sense virus of typically 15,948 nt. The *morbillivirus* group (Fig 1) includes a number of closely related viruses which infect a diverse range of hosts, of particular note are measles and canine distemper virus, which are major pathogens of humans and domesticated dogs, respectively.

**Fig 2:**
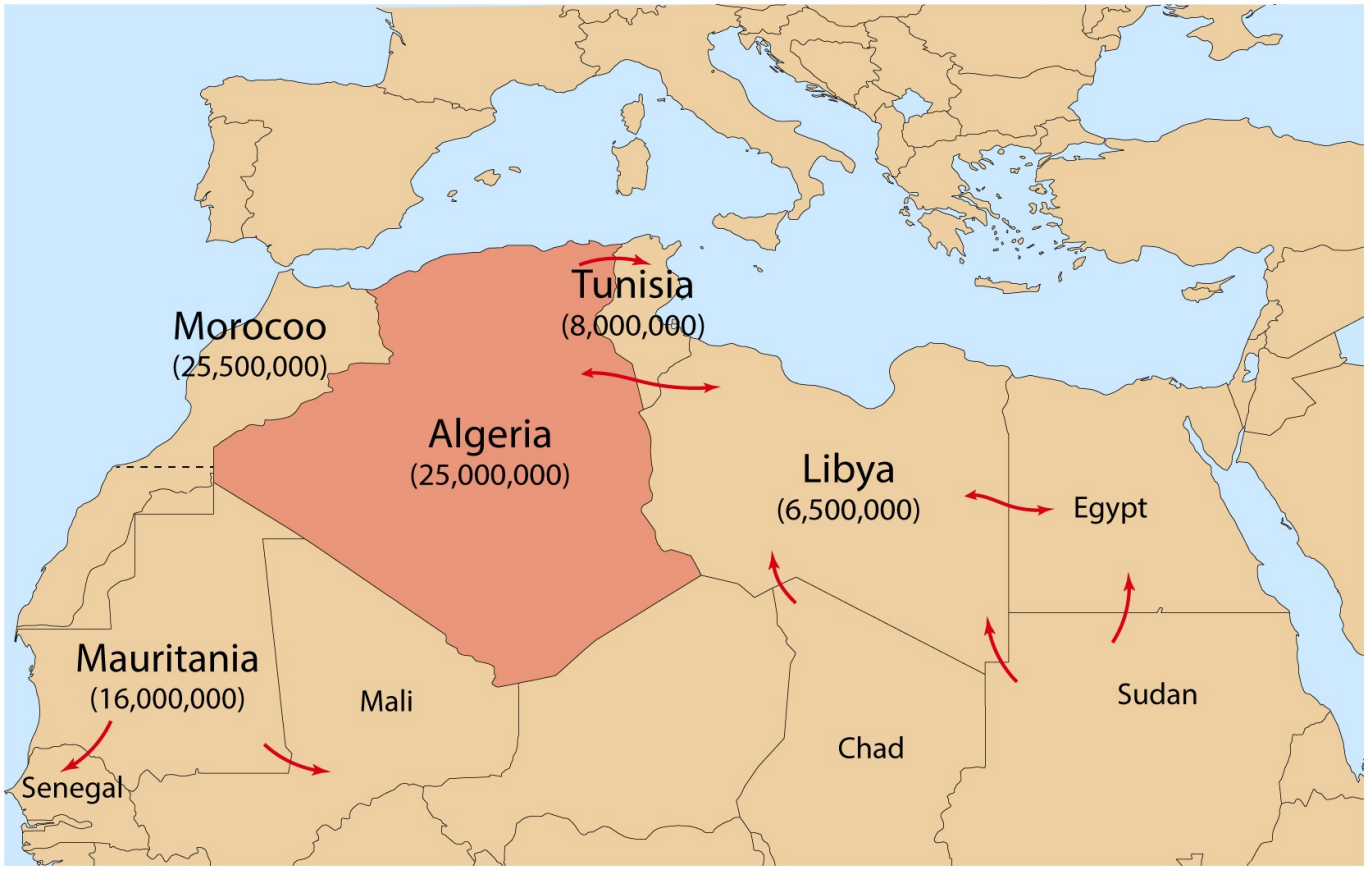
Major pathways of traditional movement of small ruminants in the Maghreb region. Pathways are indicated by red arrows and 2015 estimated populations of sheep and goats are indicated in brackets beneath Maghreb region country names. Modified from ([4, 8]).

Despite declarations by the FAO and OIE of a 2030 target for PPRV eradication [3], the spread of PPR has been facilitated by inconsistent or very restricted vaccination strategies as well as porous borders of neighboring countries between which there is significant illegal cross border animal trade through longstanding traditional animal trading routes (Fig 2) [4, 5]. PPRV now circulating throughout Northern, Eastern and West Africa, Asia—in particular China, as well as Central and Eurasia, the Indian subcontinent, as well as the Middle East [6]. The combination of PPRV in the European part of Turkey and susceptibility of deer ranging through PPR positive areas into Europe proper is cause for substantial concern for the introduction of PPR into Europe [6]. Why the spread of PPRV has dramatically increased since its detection in 1942 is yet to be determined, however, several factors are thought to have played a role. Amongst these are the lack of cross protection following the cessation of rinderpest vaccination; inconsistent and uncoordinated vaccination strategies; as well as neglect by national governments and research groups. Greater than 63% of the small ruminants globally remain under threat by PPRV [6, 7].

While the host range for morbilliviruses such as PPRV has typically been considered highly specific recently there have been a number of studies which have isolated PPRV from a more diverse range of hosts [9, 10]. In particular, PPRV specific antibodies have been detected in both domesticated and wild cattle [11] and buffalo [12, 13], gazelle, antelopes [13], Ibex [14]. Additionally, and of concern for the containment of PPRV is the recent detection of PPRV in the carnivore species Asiatic Lion [15] and dogs [16]. The devastating consequences of PPRV infection in a susceptible species outside of sheep and goats became particularly apparent in Sudan in 2010 where over 500 healthy camels died with mortality rates approaching 50% in some herds [17]. Morbilliviruses such as PPRV replicate extensively within lymphoid and epithelial cells and the signaling lymphocyte activation molecule (SLAM) is a well-established receptor for morbillivirus infection [18]. However, recently the Nectin-4 protein has been identified as a second important receptor for measles [19], canine distemper virus [20], and PPRV [18]. Both of these membrane bound receptors are highly conserved among the mammals, indeed studies have demonstrated that the canine distemper virus H glycoprotein can utilize to and infect cells using human Nectin-4 and only a single amino acid mutation 540 Asp to Gly in the receptor, is required for canine distemper virus to utilize human SLAM [21]. How effective the PPRV H gene may be at utilizing new host receptors is unknown, yet, however the conservation of the receptor molecules and PPRV H genes as well as the already wide array of hosts suggests PPRV could be an emerging threat to other species outside domesticated small ruminants.

The rapid spread of PPRV across continents particularly in North and East Africa, and Asia is a challenging environment to work towards the eradication of PPRV before 2030. These challenges are typified by the reoccurring outbreaks in the Maghreb region of North Africa (Libya, Tunisia [22, 23], Algeria [24, 25], Morocco [26–28], Mauritania, and the Western Sahara). Mass vaccination following the initial identification of PPRV in Morocco in 2008 resulted in the apparent elimination of PPRV from this country. Once these vaccination strategies were relaxed; in 2015 the OIE was notified of the reemergence of PPRV in Morocco [27].

Algeria, a North African country in the Maghreb region, is the largest African country by area. Periodic outbreaks of PPR have been reported in the south and central parts of the country since 2010 (10–11) however, the epidemiology of PPRV in Algeria has not been well studied. PPR has been reported in neighbouring countries like Morocco (2008, 2015), and Tunisia (2012–13) [23]. Following the report of lineage IV PPRV circulation in Morocco in 2008, investigations were implemented in Algerian farms and livestock markets, mainly in the western and eastern part bordering Morocco and Tunisia. In February 2012 outbreaks of PPR in Ghardaia, central Algeria (lineage IV) were reported, which shared 99% homology with the viral sequence from Tunisia [29].

This study reports on PPRV outbreaks in two farms in Cheraga, Northern Algeria in 2015, close to the Mediterranean Sea using qRT-PCR, and following full genome sequencing of the outbreak strain compares this virus to circulating PPRV within North and East Africa as well as globally using Bayesian spatial-temporal analysis. The flow of PPRV from East to North Africa following known routes of animal movement, and the persistence of PPRV within the Maghreb Region following the absence of region wide control measures and the risks to Europe following the encroachment of PPRV to the North shore of Africa are also discussed.

## Materials and methods

### Study area and sample collection

**Fig 3:**
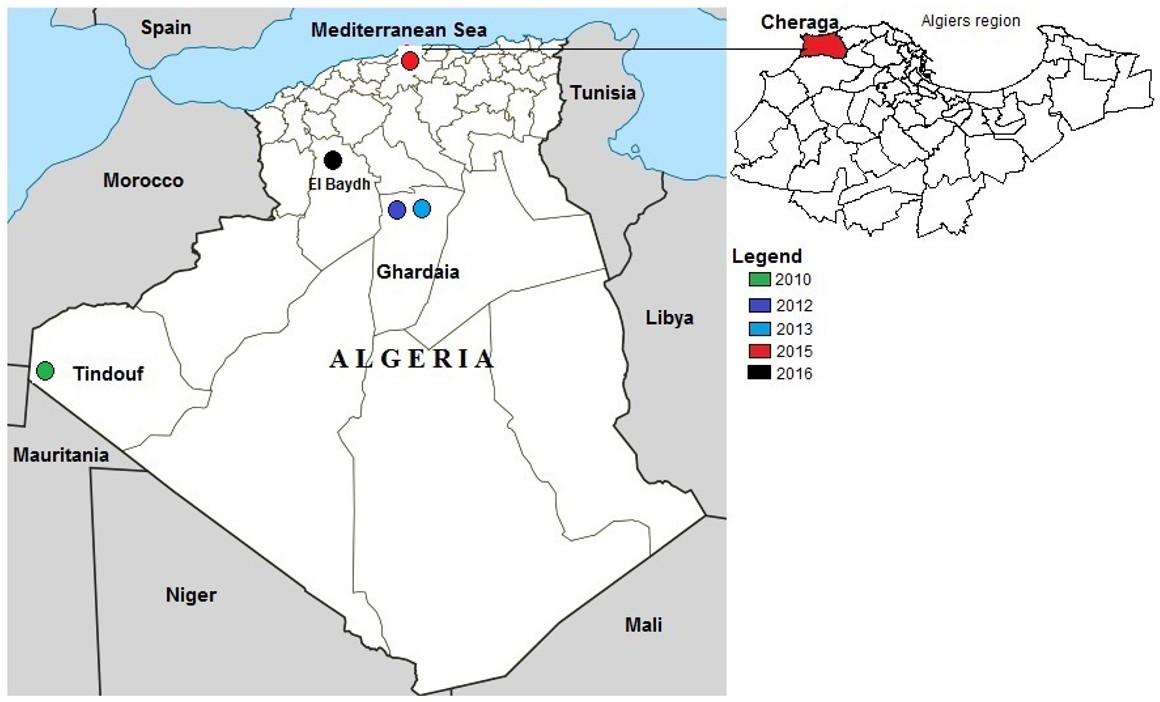
Map of locations of PPRV outbreaks in Algeria since 2010. Colours indicate outbreaks by year.

On the 23^rd^ of October 2015, local veterinary authorities in Algeria visited a suspected PPR outbreak farm (Farm 1) and a neighboring (100 meters apart) uninfected farm (Farm 2) in the Cheraga province (coordinates 36°46′00″N 2°57′00″E), a suburb of the city of Algiers in Northern Algeria (Fig 3), which practice mixed farming of sheep and goats. The farm one had 16 number of sheep and goats whereas Farm two had 25 sheep and goats in barn. On the 5th of November 2015 the outbreak was spread to the Farm two. According to the farm owner (owns both the farms) the animals were not vaccinated previously. During the religious festival of *Eid al Adha* in 2015 there was a large movement of animals in the country. One goat was introduced to Farm1 just before the festival and two goats were introduced to Farm 2 immediately after the festival. Farm one and Farm two were visited on the 23^rd^ and 25^th^ of October and the 5^th^ and 11^th^ of November 2015 and clinical examinations performed. Selected heparinized blood and tissue samples (mesenteric lymph node) from the affected animals in Farm 1 (n = 9) and Farm 2 (n = 8) (Table 1) were shipped on dry ice to The Pirbright Institute, UK for molecular analysis. Samples were collected under the usual veterinary service work in Algeria to diagnose the disease as a part of routine diagnostic activity and sent to The Pirbright Institute, UK for further diagnosis and molecular characterization.

**Table 1:** Descriptive PPR data and samples used in this study.

Date of visit	Clinical signs	Mortality* (abortion)	Samples collected for lab diagnosis
*23/10/2015*			
Farm 1	Fever, inappetence, nasal discharges, cachexia	0/16	8 total blood (goats)
Farm 2	No signs	0/25	0 samples
*25/10/2015*			
Farm 1	Fever, inappetence, occular and nasal discharges, cachexia	1 (pregnant, cachexia) /16	0 samples
Farm 2	No signs	0/25	0 samples
*05/11/2015*			
Farm 1	Nasal discharges	0/15	1 total blood (goat)
Farm 2	Fever, occular and nasal discharges	0/25	8 total blood (4 goats and 4 sheep)
*11/11/2015*			
Farm 1	Occular and nasal discharges, cahchexia; one female goat (2 years old) presented emaciation, one Sheep presented fever	0/15	15 sera
Farm 2	Fever, discharges (nasal and ocular): 4 goat presented discharge, 2 sheep (9 months and three years) presented high fever; Observation: a goat had an abortion (twin gestation) the kids were full term.	0/25 (1/25)	25 sera

* indicates number of deaths over total number of animals in the farm. Figures in parenthesis indicate number of abortions in the farm.

#### Ethical statement

Samples were collected for the diagnosis of the disease under the usual veterinary service work in Algeria, no permits were required for collection and all collections were performed according to the national standards within Algeria. The samples were sent to the Pirbright Institute (holds the PPR reference laboratory) (Import permit AHZ/1309/A) for further diagnosis and molecular characterization. When consulted, local Pirbright animal welfare ethical review board (AWERB) confirmed that no requirement of approval needed as the samples were collected primarily for veterinary diagnosis purpose at Algeria and not for the research. Further the tissue samples were collected form dead animals only.

### RNA extraction and real time reverse transcription—Polymerase chain reaction

All clinical samples were screened for the presence of viral genome by qRT-PCR. Total RNA was extracted from the clinical samples using Trizol^™^ (Invitrogen) as per manufacturer’s instruction. The presence of PPRV nucleic acids in tissue or blood samples was determined by real time RT—PCR following the method of Batten and colleagues (2011) [30]. Only qRT-PCR positive samples were taken forward for virus isolation and sequencing. Superscript III Platinum R one step qRT-PCR system kit (Invitrogen) was used for qRT-PCR.

### Polymerase Chain Reaction (PCR) and nucleotide sequencing

The viral RNA was reverse transcribed using superscript III first strand synthesis kit (Invitrogen). The C-terminus of the N-gene was amplified using primer pairs NP3/NP4 [31]. Similarly the partial F-gene was amplified using primer pairs F1/F2 [32]. The PCR amplicons were purified using the QIAEXII PCR purification kit (Qiagen) according to the manufacturer’s instructions and sequenced using BigDye^®^ Terminator v3.1 Cycle Sequencing Kit (Applied Biosystems, Carlsbad, CA, USA) using the PCR primers. Sequences (from the ABI 3730 machine) were assembled and analysed using SeqMan II (DNAStar Lasergene 8.0). Nucleotide sequences of the viruses were aligned using the CLUSTAL X multiple sequence alignment program [33].

Full length genomic RNA sequencing was performed using a hemi-nested RT-PCR as described previously [34]. Initial first-strand synthesis and first round PCR was performed as above except first-strand synthesis was performed at 48°C and an annealing temperature of 55°C was used. Second round amplification was accomplished using KOD hot-start polymerase kit (Novagen). Sequencing of the terminal 5`and 3`ends of the PPRV genome was accomplished via RACE, as previously described [34].

### Virus isolation

In addition to Trizol^™^ RNA isolation, attempts were made to isolate virus from tissue samples. Briefly, 5 cm^3^ tissue pieces were homogenised in Buffer M25 (0.04 phosphate buffer, disodium hydrogen phosphate, potassium dihydrogen phosphate, with 25 mM NaOH pH 7.5) using a mortar and pestle. The homogenate was clarified by centrifugation at 3,000 rpm for 15 minutes at +4°C and 500 μl of the supernatant were inoculated onto 70% confluent Vero dog slam cells (VDS) and incubated for 2 hours at 37°C in an atmosphere of 5 per cent CO_2_, before inoculant were replaced with Dulbecco's Modified Eagle's medium (DMEM) supplemented with 5% foetal calf serum (FCS) and penicillin and streptomycin. Following incubation cells were incubated for up to 7 days and blind passaged 3 times or until cytopathic effects CPE were observed.

### Sequence datasets and temporal phylogenetics

For sequence data not generated in this study, representative partial N gene sequences, genome position 1360–1614 (255 nt) (S1 Table), as well as complete PPRV genome sequences (S2 Table) were obtained from GenBank. The partial N dataset was aligned using MUSCLE and phylogenetic analyses were performed using MEGA6 [35]. The neighbour-joining tree was computed using the Kimura 2-parameter model and tests for phylogeny performed using the bootstrap method with 5000 replications and the gaps/missing data treated with pairwise deletion [36].

To identify the nearest common ancestor and hence likely dates of divergence, the Algeria/Cheraga/2015 (Accession Number KY885100) sequence was compared using the coalescent-based Bayesian Markov chain Monte Carlo (MCMC) [37, 38] approach to all available full length PPRV wild type genomes available in the GenBank (n = 41; 22/10/2016). To ensure that high passage number vaccine strain sequence data did not skew the results as has been previously demonstrated [39], these sequences were removed from the following analysis (n = 37) (India/Sungri 96: KJ867542, KF727981 and Nigeria 75 (X74443, HQ197753). The general time-reversible nucleotide substitution model with a gamma distribution for rate variation and proportion of invariant sites was selected on the basis of Akaike information criterion scores. Previous Bayes factor tests with smaller numbers of complete genomes (n = 14) have utilised the relaxed uncorrelated exponential distribution (UCED) clock model [40] as the best fit to PPRV complete genomes [39, 41] and Bayes factor tests [37, 38] with marginal likelihood comparisons showed that this remained the case with the larger dataset.

### Geospatial phylogeny

Bayesian phylogeographic analysis of complete full length sequences [42] were performed by using complete PPRV genome sequence isolates and annotated according to their location (longitude and latitude); where possible provincial level location data was used otherwise country centre details were utilized. Latitude and longitudinal information was extracted from the google maps API. The same 37 virus isolates as well as the new Algeria/ Cheraga/2015 as per temporal phylogeny was utilized. Phylogeographic diffusion along the posterior sets of trees and relationships between these locations were identified by using the Bayesian stochastic search variable selection procedure in BEAST v1.8.4 [42]. Discrete phylogeographic analysis was performed by using the continuous time Markov chain with the flexible Bayesian skyride tree prior [43–47]. To further investigate the special-temporal relationship between North and Eastern African Lineage IV viruses a further set of all partial N sequences available from Genbank (S3 Table) were added to virus sequences isolated in this study. As above model selection indicated that the general time-reversible nucleotide substitution model with a gamma distribution for rate variation and UCED relaxed clock with coalescent exponential growth was the most appropriate model for this analysis. All Bayesian analyses were run for 10,000,000 iterations sampled every 10,000 in duplicate; duplicate runs were combined for final analysis, ESS values >200 were obtained for all indicators following combination.

### Ethics statements

As the samples were collected for the diagnosis of the disease under the usual veterinary service work in Algeria no permits were required for collection. The samples were sent to the Pirbright Institute (hosts the PPR reference laboratory) for further diagnosis and molecular characterisation.

## Results and discussion

### Field observations

The animals of Farm 1 (n = 16) exhibited typical signs of PPR: inappetence, fever (> 40°C), and ocular and nasal discharges. In addition, diarrhoea and bronchopneumonia were observed (Table 1). On the 25^th^ October 2015, the first mortality, a four-year-old pregnant goat exhibiting signs of cachexia and diarrhoea in Farm 1 was reported. However no clinical symptoms were reported at Farm 2 on either the 23^rd^ or 25^th^ of November. On the 5th of November 2015 an additional outbreak was reported at Farm two in a barn (n = 25). A female goat in Farm 2 aborted twins late in gestation on the 11^th^ of November 2015. Detailed clinical examination and samples collections were described in Tables 1 and 2.

**Table 2:** Details of samples collected and PCR test results.

Animal No.	Species	Sex	Age	F/RT-PCR^#^	N/RT-PCR	qPCR
*Farm 1*						
1	Caprine	F	9 months	-	-	+
2	Caprine	M	7 months	-	+	+
3	Caprine	M	9 months	-	-	+
4	Caprine	F	4 years	-	-	-
5	Caprine	M	9 months	-	-	+
6	Caprine	F	5 years	-	+	+
7	Caprine	F	5 years	-	+	+
8	Caprine	F	2.5 years	-	-	+
9	Caprine	M	7 months	+	+	+
*Farm 2*						
1	Caprine	F	1 year	+	-	+
2	Ovine	F	3 years	+	-	+
3	Caprine	F	8.5 months	+	+	+
4	Caprine	F	1 year	+	+	+
5	Caprine	M	9 months	+	-	+
6	Ovine	M	6 months	-	-	-
7	Ovine	M	3 years	+	-	-
8	Ovine	F	9 months	+	+	+

^#^ indicates nested PCR

This is the first report of PPR in Algeria since the 2013 outbreaks and the most northern. Prior to this outbreak, Algeria has notified the OIE of PPR three times since 2010 (Fig 3). Outbreaks were highlighted in the South in 2010 and in central Algeria (Ghardaia) in 2012 and 2013 (Fig 2). Following the outbreak in Ghardaia, vaccinations were performed in 118797 goats in provinces neighboring Ghardaia. Outbreaks in Cheraga, Algiers (October 2015) as detailed in this study, and North-West Morocco (June, 2015) [48] showed that PPRV has spread to the north of these countries, this further spread may pose a threat of introduction to Europe.

### Molecular analysis and sequencing

**Fig 4:**
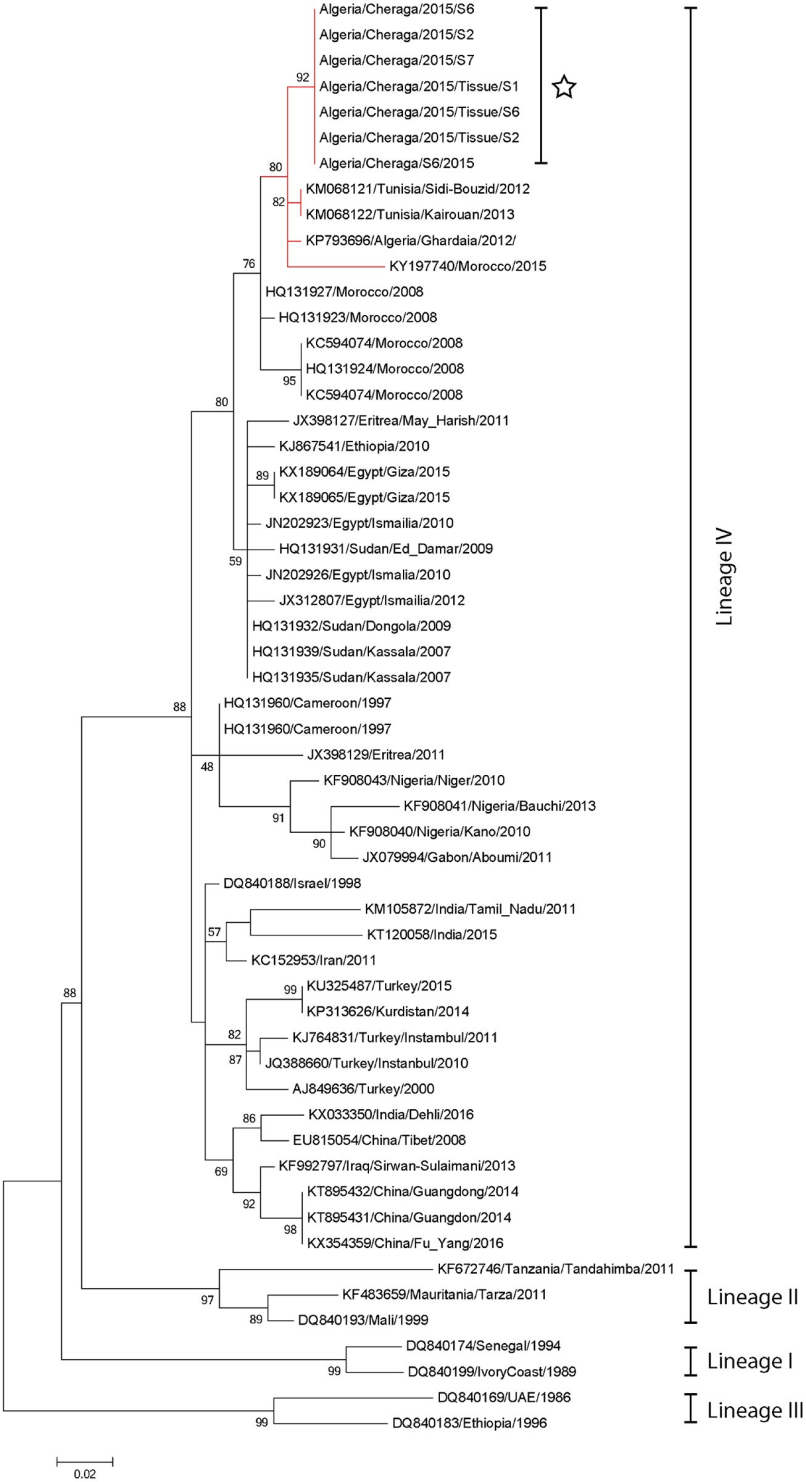
Neighbor-joining tree based on a 255 nt partial PPRV N gene sequence (1360–1614 nt) of peste des petits ruminants virus isolated from a 2015 Algerian outbreak (White star) (KY885101, KY885102, KY885103) and selected sequences from GenBank. Nodes are labeled with bootstrap values obtained following 5000 replications with the Kimura 2-parameter model. GenBank accession numbers are given against each sequence. Scale bar indicates nucleotide substitutions per site. Branches leading to isolates which are obtained from the Maghreb region of North Africa are labeled in red.

Eight blood samples from the first outbreak and seven from the second outbreak were positive by qRT-PCR with Ct-values ranging from 11.4 to 33.5, indicating a high viral load. Four samples collected from the Farm 1 and three samples from Farm 2 were positive in N-gene specific gel-based PCR (Table 2), four of the six tissue samples from Farm2 were also positive in N-gene PCR. One blood sample from Farm 1 and two blood samples from Farm 2 were used for sequencing partial N sequencing (Accession Nubmers KY885101, KY885102, KY885103), in addition to all four positive tissues. All sequences were 100% identical and phylogenic analysis grouped these isolates with the lineage IV viruses. Further analysis showed that the Algeria/Cheraga/2015 isolates form a distinct clade with the viruses isolated from the 2012 outbreak in Ghardaia, Algeria; and the 2012 and 2013 outbreaks in Tunisia as well as the 2015 virus recently sequenced from the 2015 outbreak in Morocco. The sequences isolated previously from the 2008 Moroccan outbreak grouped together within the same obvious clade however they were distinct from the more recent samples, suggesting the currently circulating Algerian and Tunisian virus has migrated into Morocco during 2015 (Fig 4). In addition, partial N gene analysis clearly showed 3 distinct clades of lineage IV viruses in circulation, East and North African clade, West and central African clade and an Asian and Middle East clade. The North and East African clade was further divided into two clear separate groups.

In addition to sequencing the partial N variable region, full length sequencing was undertaken of RNA harvested from one of the tissues obtained from Farm 2. As expected the genome of the Algeria/Cheraga/2015 virus isolate was 15,948 nt and conformed to the rule of 6. The genomic structure of the virus was as expected, including intergenic CTT motifs, gene start and end sites, and non-coding leader and trailer regions. Comparisons of the full length genome between the KY885100/Algeria/Cheraga/2015 isolate and the most closely related KC594074/Morocco/2008, KJ867541/Ethiopia/2010 confirmed that these viruses are greater than 98% identical. Unfortunately the full genome sequences of 2012 Algeria virus (KP793696), and the 2012 (KM068121) and 2013 (KM068122) Tunisia viruses and 2015 Morocco virus (KY197740) are not available. However based on the partial N gene sequences the closest relationship has been seen with 2012 Algerian (98.8%), and 2012 and 2013 Tunisian isolates (98.8%) suggesting the continual circulation of this virus in Algeria as well as in the surrounding region.

### Temporal—Spatial spread of PPRV

**Fig 5:**
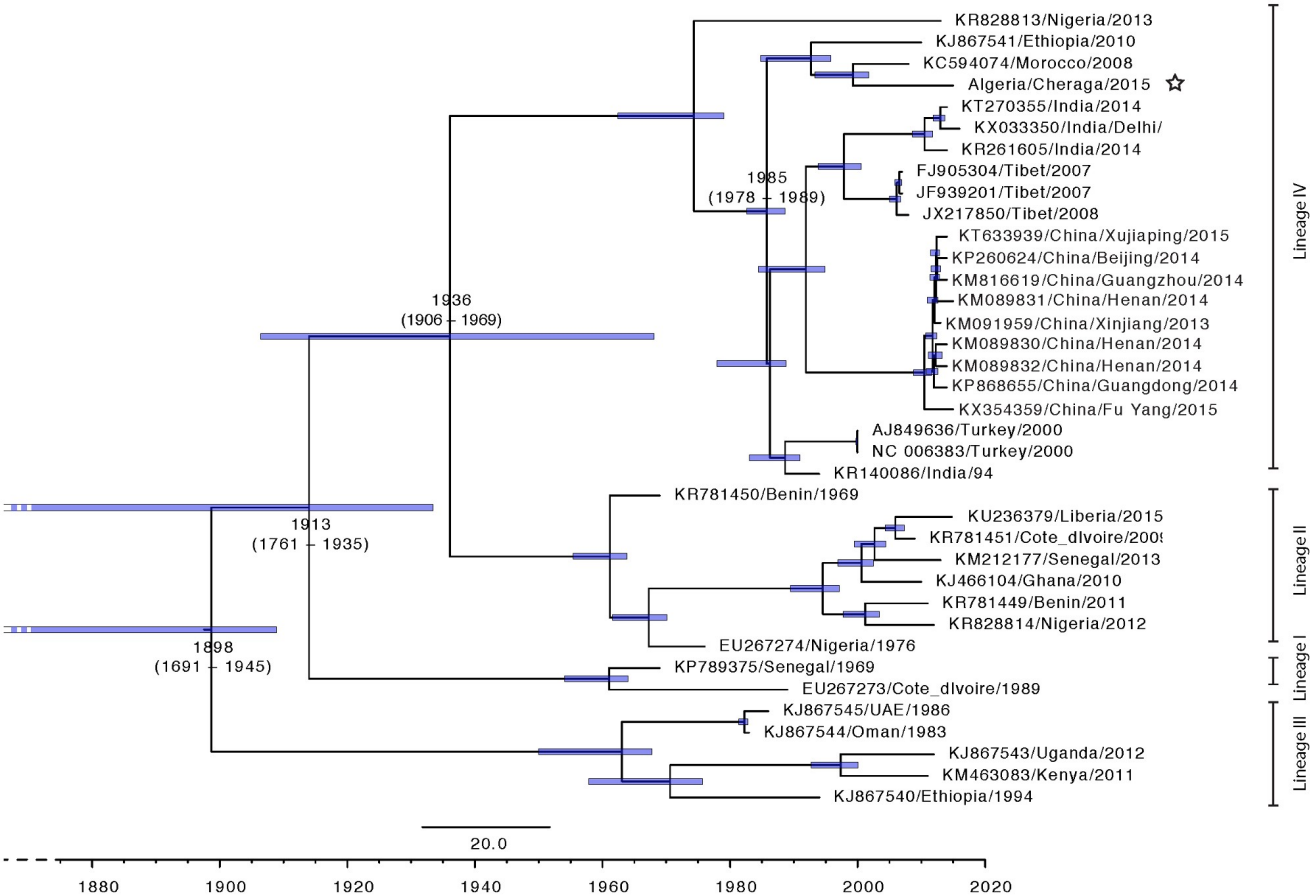
Time-scaled Bayesian MCC phylogeny tree based on peste des petits ruminants virus complete genome sequences (n = 38). The Algeria/Cheraga/2015 sequence is indicated by a white star. The tree was constructed using the uncorrelated exponential distribution model and exponential tree prior. Branch tips locations correspond to year of collection and tip labels indicate accession number, as well as location and year of collection and branch lengths reflect elapsed time. Tree nodes were annotated with the estimated median time to most recent common ancestor (TMRCA). Corresponding 95% highest posterior density (HPD) values of TMRCA are indicated as blue bars. Horizontal axis indicates time in years and for presentation truncated to 1860.

Using the established models, a Bayesian time-scaled MCC maximum likelihood tree was constructed for all (n = 37) available PPRV full length genomes and the Algeria/Cheraga/2015 PPRV virus (Fig 5). The estimated time of divergence of this group of viruses from other circulating lineage IV viruses is median TMRCA = 1985 (95% HPD 1978–1989). Other notable times include the median TMRCA at the divergence of lineage III for all included virus strains is 1898 (95% HPD 1691–1945), Lineage I diverged in 1913 (95% HPD 1761–1935), and Lineages II and IV diverged in 1936 (95% HPD 1906–1969) (Fig 5). These remain broadly comparable with previous analyses [39, 41], however as has been noted in a number of reviews and other articles the sample bias towards recently isolated samples is likely to sharply weight the TMRCA towards being more recent than more distant.

### Phyologeography of PPRV

**Fig 6:**
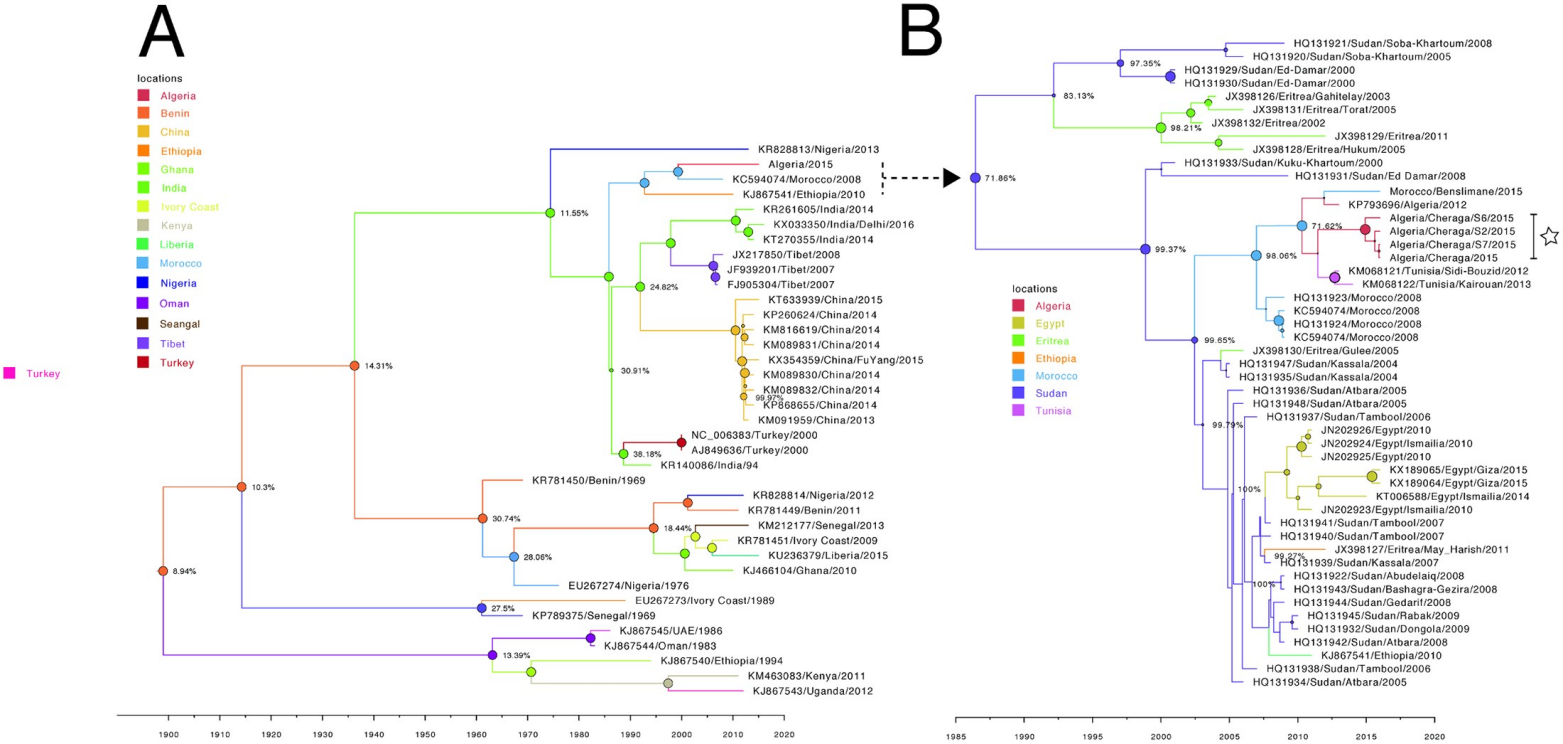
Maximum clade credibility trees constructed for the phylogeographical reconstruction of PPRV using complete genome data (A) or partial N sequences derived from North or East Africa (B). Posterior probability values are indicated by the size of the node and posterior probability distribution indicated as a percentage at the size of each node. Branches are colored according to most likely location at the preceding node. Horizontal axis indicates time in years. Samples for which have been sequenced in this study are highlighted by a white star. GenBank accession numbers are given against each sequence where available.

To estimate the route of entry of PPRV into North Africa we visualized the summarized results of the Bayesian Phylogeographic analysis of full length PPRV sequences (Fig 6A). Analysis of the posterior probabilities (Fig 6a Nodes) suggests a strong historical and geographic connection between the Maghreb region isolates (Morocco/2008, Algeria/Cheraga/2015) and viruses isolated in East Africa (Ethiopia/2010). As the available number of full-length sequences is very small, to explore the relationship between Northern and Eastern African virus isolates further phylogeographic analysis was performed using all available partial N (255 nt; between nts 1360–1614) sequences from all lineage IV Eastern African countries (Ethiopia, Sudan, Egypt, Eritrea) (Fig 6B). These analyses show a very strong likelihood >70% that the lineage IV viruses currently circulating in the Maghreb region of North Africa spread from East Africa (Sudan 2000 outbreak virus) in the first decade of twenty first century and were first reported in Morocco in 2008. The movement of small ruminants and camelids from the Sahel particularly Sudan is well recognized as a putative pathway for the spread of disease into North Africa [4, 8].

The lineage IV PPRV in North Africa appears to have sprung from East Africa following the outbreak in Sudan in the year 2000 –this outbreak has previously been characterized as splitting into two groups one affecting sheep and goats (HQ313933, HQ131931) and the second camels (HQ131929, HQ131930) [49]. These sheep and goat derived viruses appear to be from the available sequence data the most likely progenitor viruses for the closely related (97.6% identity across all viruses utilized in this analysis Fig 6B) subsequent isolates in East and North Africa. In this case the weighting of sample numbers from particular countries particularly Sudan (n = 20/40) likely over-estimates its importance in the spread of PPRV within East and North Africa. Within the same time period there are published reports of wide spread PPRV infection and serological positive isolates for PPRV in the surrounding region for which there is no sequence data available; Egypt (2006) [50], Ethiopia 2004–2010 [51, 52], and Chad [53].

## Conclusion

The results obtained in this study demonstrate that the PPR has spread in time and space and that PPR has been continuously circulating through Maghreb region since 2008, despite vaccination efforts in Morocco. Sequence analysis of the highly variable partial N region as well as of the full length PPRV genomes demonstrates the presence of the same circulating virus throughout Algeria, Morocco and Tunisia. The repeated PPR outbreaks in southern and central Algeria and the subsequent spread to northern Algeria and reintroduction of this virus into northwest Morocco, despite mass vaccination throughout Morocco and targeted vaccination in Algeria demonstrates the importance of a consistent and focused, as opposed to *ad hoc* strategy to combat PPRV across the Maghreb Region and North Africa.

The focussed implementation of strategies to combat PPR remains predominantly absent globally, despite safe vaccines which provide sterilising immunity being available for more than 25 years. Strategic regional vaccination as opposed to sporadic focused vaccination following disease outbreak has been used to remarkable effect in Morocco following the 2008 outbreak, as well as in Tibet following the 2007/2008 outbreaks and 2013–2014 large outbreaks in that province of main land of China, as well as provincially in India. However outside of these exceptions strategic region wide strategies for the control of PPRV have been neglected. The importance of regional strategies for the control of PPRV is demonstrated by the reoccurrence of the disease in El Bayadh, Central Algeria, involving both sheep and goats whilst this manuscript was being prepared in February 2016. In addition, the recurrence of the disease in Morocco in June 2015 [48] provides further evidence of the virus circulation in the region. Considering the same virus is circulating in Algeria, Morocco and Tunisia, implementation of a common Maghreb strategy to combat PPR should be undertaken as a priority and reinforces the importance of strategic widespread policies and vaccination if the 2030 target of PPRV eradication is to be reached. Until these strategies are implemented PPRV will remain the most important neglected disease of small ruminants globally.

In the light of continuous circulation of the virus in the Maghreb region of North Africa and its recent move towards the Northern part of Algeria and Morocco approaching Gibraltar and the extensive trade links with Europe (Spain, France and Italy), increased risk of introduction of PPR to Europe is a huge concern. Considering importing live animals from countries endemic for sheep and goat pox is forbidden, the movement of sheep and goats from North Africa to Europe is unlikely [6]. However, movement of live animals from Europe to the Maghreb region have been noted, particularly towards the end of religious festivals like Ramadan and the month preceding Hajj. Therefore, there is a chance of PPR spread from North African countries to Europe through fomites, potentially through returning trucks used for transport of animals to North Africa, as well as through the illegal importation of meat or live animals. Therefore proper cleaning and disinfection of vehicles transporting livestock is essential before these vehicles return to Europe, as well as strong customs and quarentine control at routine sites of entry to Europe from North Africa [6].

## Supporting information

S1 Table Full-length PPRV sequences utilized in this study retrieved 29/10/2016 from NCBI. (XLSX)

S2 Table Partial n isolates utilized in the preparation of Fig 4 retrieved from NCBI 29/10/2016. (XLSX)

S3 Table Partial n North and East African isolates utilized in the preparation of Fig 6B retrieved from NCBI and annotated via google maps 29/10/2016. (XLSX)
